# Percutaneous Endoscopic Lumbar Discectomy for Lumbar Disc Herniation Using an Endoscopic Staining: A Technical Note

**DOI:** 10.1111/os.12907

**Published:** 2021-05-04

**Authors:** Jingjing Tang, Ziyang Liang, Jiahui He, Qi Shang, Jiarui Zhang, Zhihua Wu, Xiaobing Jiang, De Liang, Hui Ren, Jianchao Cui, Zelong Zhou, Zhensong Yao

**Affiliations:** ^1^ Department of Spine Surgery the 1st Affiliated Hospital of Guangzhou University of Chinese Medicine Guangzhou China; ^2^ First Clinical Medical College Guangzhou University of Chinese Medicine Guangzhou China

**Keywords:** Decompression, Endoscopic surgery, Intervertebral disc disease, Staining

## Abstract

Symptomatic lumbar disc herniation (LDH) is widely treated using percutaneous endoscopic lumbar discectomy (PELD). In the present PELD surgery, performing decompression under endoscope still takes a long time to explore the rupture site of annulus fibrosus, resulting in prolonged operation time and over‐invasion of the undegenerated annulus fibrosus. A wide range of intraoperative exploration also induces an iatrogenic injury of the normal annulus fibrosus, even aggravating intervertebral disc degeneration, which may lead to early postoperative recurrence in severe case. Hence, it is important to seek a precise decompression in PELD surgery. Under this kind of realization, more spinal surgeons possibly choose a disc staining before performing decompression. However, the classical disc staining technique still has its shortcomings. First of all, an appropriate dose of staining cannot be accurately mastered, even induces unqualified staining effect. Second, the duration of surgery and the times of fluoroscopy will be increased. Finally, what surgeons see under the endoscope is the staining result but not the staining process. Hence, this is accomplished more effectively by designing procedures that perform fully visible disc staining under spinal endoscope. There is no specific research to discuss the technique note of endoscopic staining in PELD surgery. We have come up with a new original technology of endoscopic staining with methylene blue injection in PELD for treatment of LDH.

## Graphical abstract

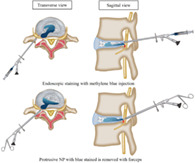

This current study is to display a series cases using a new and original endoscopic disc staining. This modified technique can fully observe the process of disc staining. It also minimizes the iatrogenic injury of the intraoperative undegenerated annulus fibrosus. In addition, the learning curve is smooth and it is easy to master this technique for experienced orthopedic and neurosurgical spine specialists.

## Introduction

To date, symptomatic lumbar disc herniation (LDH) has been widely treated using percutaneous endoscopic lumbar discectomy (PELD) because of its advantages. The PELD operation was superior in terms of tissue injury, bleeding volume and recovery period^1^, ^2^. This inception does accord with spinal surgery's basic principle, which is to treat diseases effectively with minimal structure invasion of normal anatomy. Since PELD has become a representative, minimally invasive spine surgery for LDH, there also exists limitations. Some modified procedures in the process of PELD have emerged and improved surgical solution. Li *et al*. designed protective working cannula in the original Tessys technique and used a trephine cut the bony structure of superior articular process through the tube^3^. Meanwhile, we also researched the efficacy and safety of trephine for axillary‐type LDH. Using a trephine with protective working cannula for foraminoplasty of superior articular process have shown its safety and high efficiency^4^. Hence, appropriate modification is necessary in surgical solution and possibly improves clinical outcomes. Endoscopic spine surgery has evolved gradually through improvements in endoscope design, instrumentation, and surgical techniques.

In the present PELD surgery, performing decompression under endoscope still takes a long time to explore the rupture site of annulus fibrosus, resulting in prolonged operation time and over‐invasion of the undegenerated annulus fibrosus. Sometimes the boundary between normal annulus fibrosus tissue and degenerated nucleus pulposus could not be clearly and quickly distinguished intraoperatively. So, a long intraoperative probe is inevitably required. Moreover, a wide range of intraoperative exploration will induce an iatrogenic injury of the normal annulus fibrosus, even aggravating intervertebral disc degeneration, which may lead to early postoperative recurrence in severe cases. Undegenerated annulus fibrosus prevents the nucleus pulposus from herniating or leaking out of the disc by sealing the nucleus and evenly distributing any pressure and force imposed on the intervertebral disc^5^. Based on this reason, some studies considered it will lead to an iatrogenic injury^6^ and increase the risk of early postoperative recurrence^7^. So, it is important to seek a precise decompression in PELD surgery. Under this kind of realization, more spinal surgeons possibly choose a disc staining before performing decompression^8^, ^9^, ^10^. Moreover, disc staining can better distinguish between a nucleus pulposus and a nerve root, and decrease the risk of injury to the exiting and traversing nerve roots. The technique of disc staining can date back to the 1980s. Schreiber *et al*. and Suezawa *et al*. published their bilateral approach for a percutaneous nucleotomy under endoscopic control and described injecting indigo carmine into the disc space to stain the abnormal nucleus pulposus and annulus fibrosus^11^, ^12^, ^13^. This is based on a strong relationship for usefulness of the application of methylene blue for selective endoscopic intervertebral nuclectomy in degenerated nucleus. Kim *et al*. have demonstrated that methylene blue is highly reactive with acidic extracellular matrix in the degenerated nucleus pulposus^14^.

A classical disc staining technique is performed by injecting methylene blue into the disc in accordance with the puncture approach of discography. He *et al*. performed a chromo‐discography using a mixture of iohexol and methylene blue to disc, inducing less chance of iatrogenic lumbar instability and the formation of intracanal scar tissue^15^. Several studies involved large numbers of cases have shown that the disc staining technique has become common for PELD surgery^16^, ^17^. However, this classical technique still has its shortcomings. First of all, an appropriate dose of staining cannot be accurately mastered and can even induce unqualified staining effect. Second, the duration of surgery and the times of fluoroscopy will be increased. Finally, what surgeons see under the endoscope is the staining result but not the staining process. Hence, this is being accomplished more effectively by designing procedures that perform fully visible disc staining under a spinal endoscope.

The aim of this work is to display a series of cases using a new and original endoscopic disc staining. To our knowledge, there is no specific research to discuss the technique note of endoscopic staining in PELD surgery. This modified technique not only recognizes stained nucleus pulposus, but also fully observes the process of disc staining. Our strategy also avoids the excessive removal of the nucleus pulposus and minimizes the iatrogenic injury of the intraoperative undegenerated annulus fibrosus, to help maintain long‐term disc function in the movement of the spinal functional unit.

## Case Presentation and Surgical Technique

**Fig 1:**
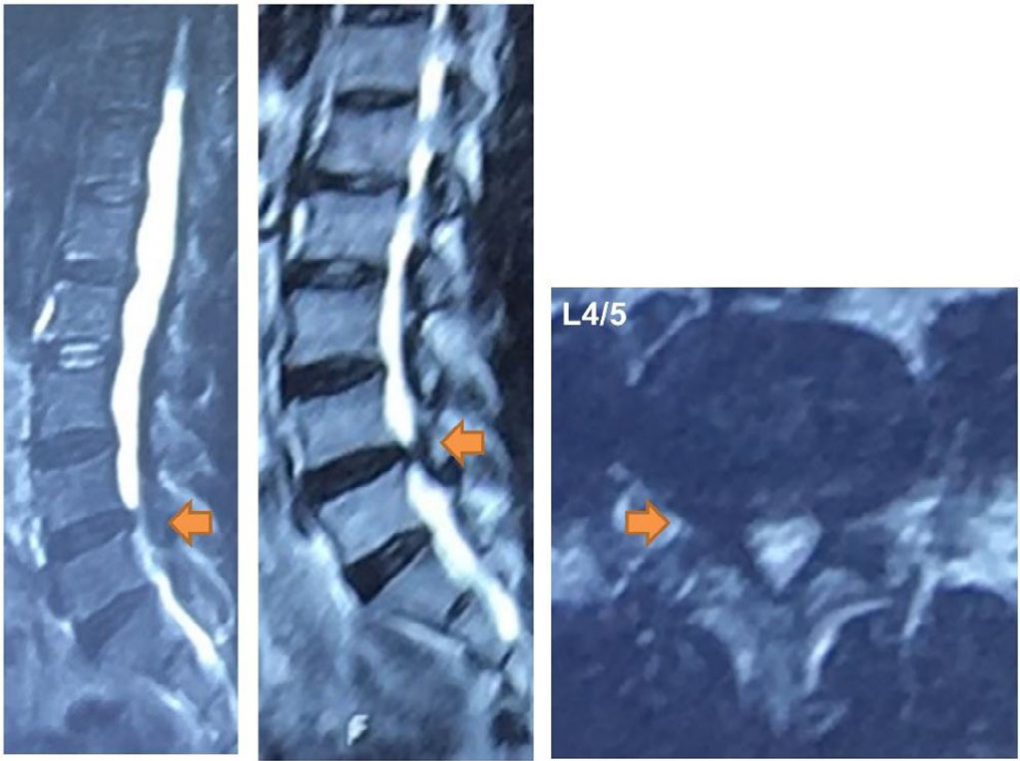
Preoperative MRI revealed herniated disc compression on the right nerve root and right lateral recess stenosis at L4‐5.

A 50‐year‐old male presented with 6 months of radiating pain and numb to right leg. Physical examination revealed a positive straight leg raising test at 40°on the right side. There was weakness of the ankle and great toe dorsiflexion (muscle strength graded IV and III, respectively). MRI revealed a herniated disc compressed on the right nerve root and right lateral recess stenosis at L4‐5 (Fig. 1). The surgical protocol was set as L4/5 PELD.

**Fig 2:**
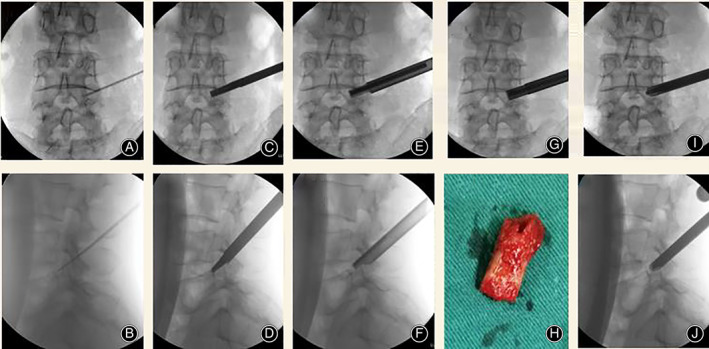
Foraminoplasty process in PELD surgery. (A, B) A puncture needle is inserted into the intervertebral foramen of L4/5; (C, D) After infiltrating 20 ml of 0.5% lidocaine into the intervertebral foramen, the needle was replaced with a tapered cannulated guide rod, which was stuck into the intervertebral foramen; (E, F) Following the guide rod, the protective sleeve accompanied with trephine was inserted, and with the trephine; (G, H) Parts of the bone on the ventral and the cuspidate articular process were abraded with the trephine; (I, J) Taking out trephine, the working sleeve is inserted into the intervertebral foramen for establishing a working channel.

**Fig 3:**
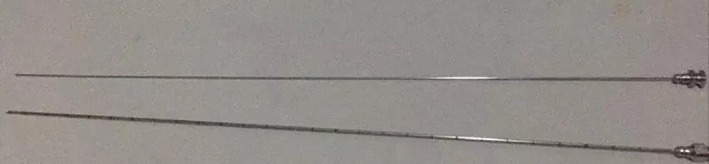
Once the surface of operative disc is clearly visible under the spinal endoscope, using a custom‐made injectable needle inserted into the disc.

After the general anesthesia, the patient was placed in a prone position on a soft cushion for spinal surgery. C‐arm fluoroscopy was used for anteroposterior and lateral view to ensure L4‐5 intervertebral space. The position of the fluoroscope and the height of the operating table should be checked for convenience for the operating team. The patient was marked for puncture orientation and prepped and draped in standard sterile procedure. 0.5 wt% of lidocaine was used for local infiltration anesthesia around the skin, fascia, and the facet joint. Aided by C‐arm fluoroscopy, a puncture needle is inserted into the intervertebral foramen of L4/5 by layers. The tip was confirmed in the anteroposterior position at the inner margin of the vertebral pedicle, while the lateral position was in the posterior and upper margin of vertebral pedicle of L5 (Fig. 2A,B). After the intraformational infiltration anesthesia was completed in the right intervertebral foramen, the guide wire was inserted along the puncture needle. A 7‐mm cut was made in the center of the puncture point on the skin. The guide wire was inserted along a tapered cannulated dilator to enlarge surgical access, as well as an ongoing dialogue with the patient. The working sleeve was implanted along tapered cannulated dilator to separate the surrounding soft tissue (Fig. 2C,D), then the protective sleeve of trephine was also inserted. The soft tissue was expanded step by step using the protective sleeve until its tip was at medial edge of the vertebral pedicle in anteroposterior view and posterior edge of the vertebral body in lateral view (Fig. 2E,F)., respectively. Afterwards, parts of the bone on the ventral and the cuspidate articular process were abraded with the trephine (Fig. 2G,H), and the intervertebral foramen was expanded to form a passageway, then the working sleeve was inserted to the previous position (Fig. 2I,J). Next, the surgeon connected the video endoscope and confirmed that the endoscope to be working. Bipolar electrocautery was used to obtain meticulous hemostasis and nerve detacher carefully separated ligamentum flavum to expose part of the normal annulus. A long syringe (Spinendos Co, Germany) was punctured into the intervertebral disc under the direct use of the endoscope (Fig. 3).

**Fig 4:**
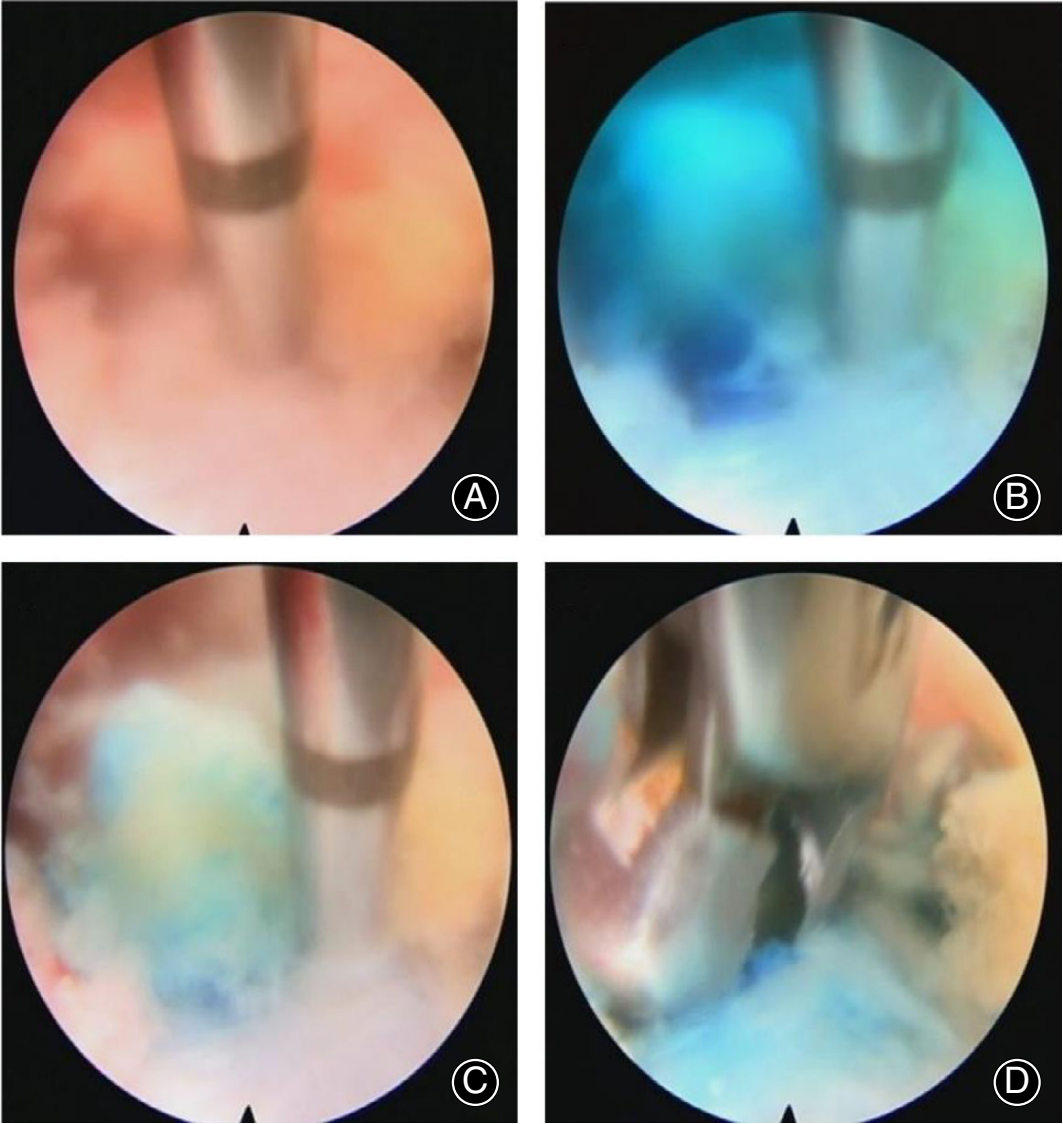
Endoscopic staining with methylene blue injection and subsequent removal of the nucleus pulposus. (A) A long syringe was punctured into the intervertebral disc under the direct vision of the endoscope. (B) Methylene blue mixture was injected; (C) Nucleus pulposus was rushed out due to the increased pressure in disc; (D) Protrusive NP was removed with forceps.

**Fig 5:**
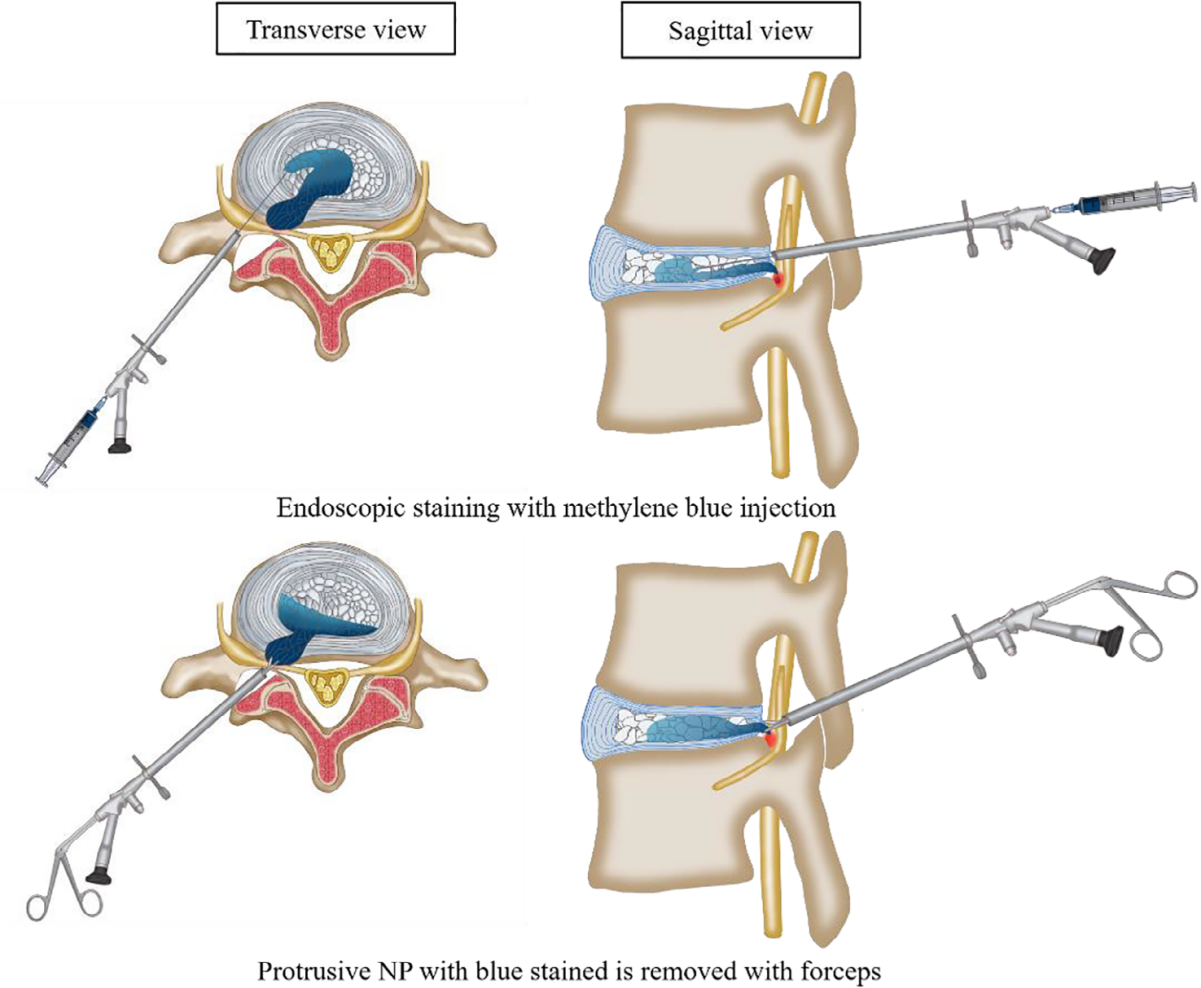
Surgical diagrams for endoscopic staining in PELD surgery.

**Fig 6:**
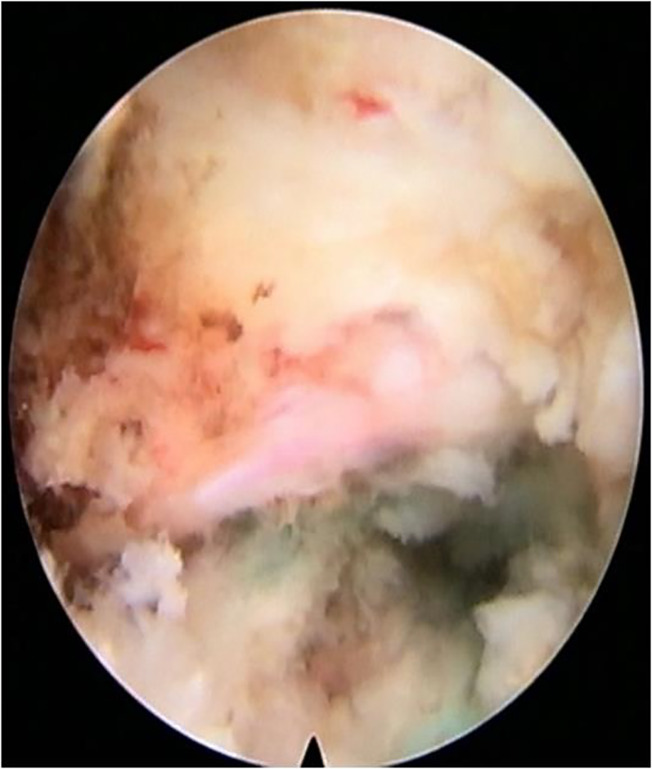
Disc herniation was successfully decompressed in the endoscopic view.

**Fig 7:**
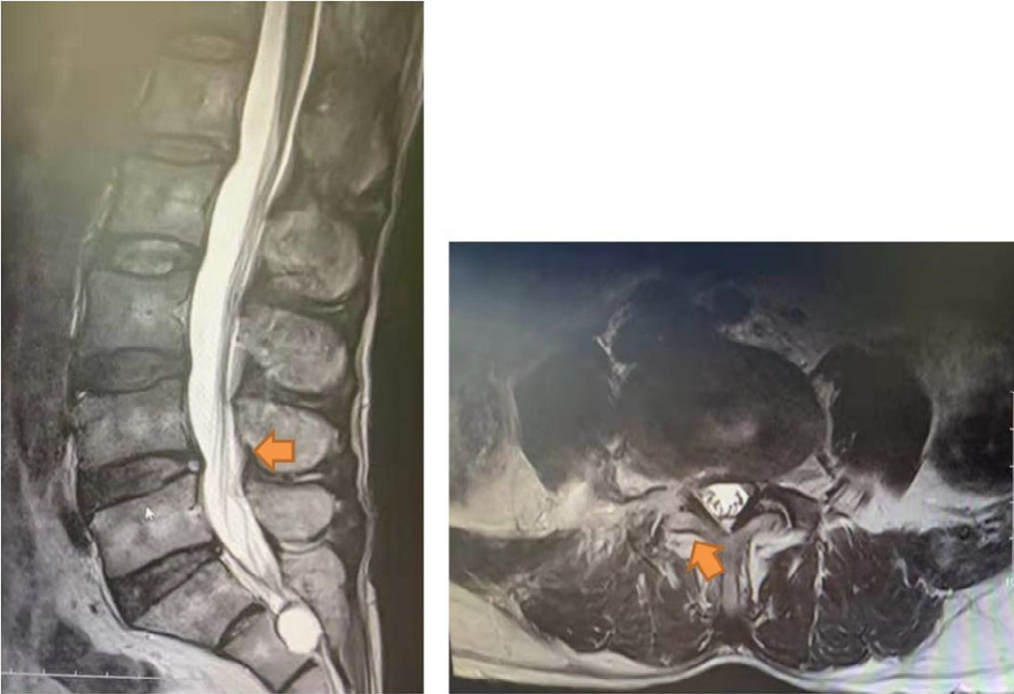
A postoperative MRI confirmed that the disc herniation was successfully decompressed, foraminoplasty can also be seen in the postoperative MRI.

**Fig 8:**
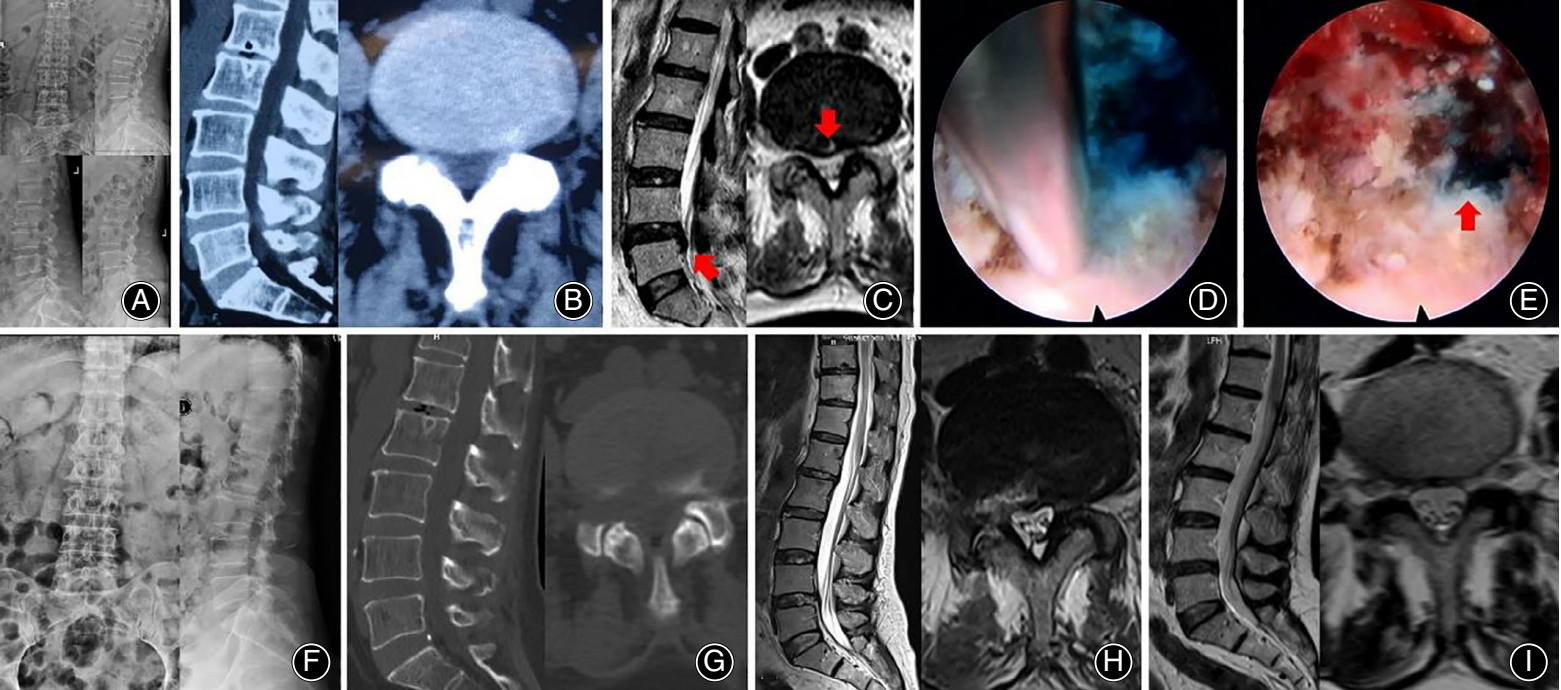
A 58‐year‐old female patient suffered from right lower extremity pain for 3 months and underwent L4‐5 PELD surgery. (A, B, and C) Preoperative lumbar spine X‐ray, CT, and MRI. Her diagnosis was L4‐5 LDH (arrow). (D and E) The long syringe is punctured into the intervertebral disc under the endoscopic view and the methylene blue mixture is injected. The rupture of the annulus fibrosus appears under endoscope. (F, G, and H) Postoperative lumbar spine X‐ray, CT, and MRI. MRI showed the herniated disc has been removed completely. Postoperative ODI value 10% value and VAS score (0 score) were significantly lower than preoperative ODI (48%) and VAS (5 score). (I) Lumbar MRI images at 6 months follow‐up showed annulus fibrosus self‐rehabilitation.

**Fig 9:**
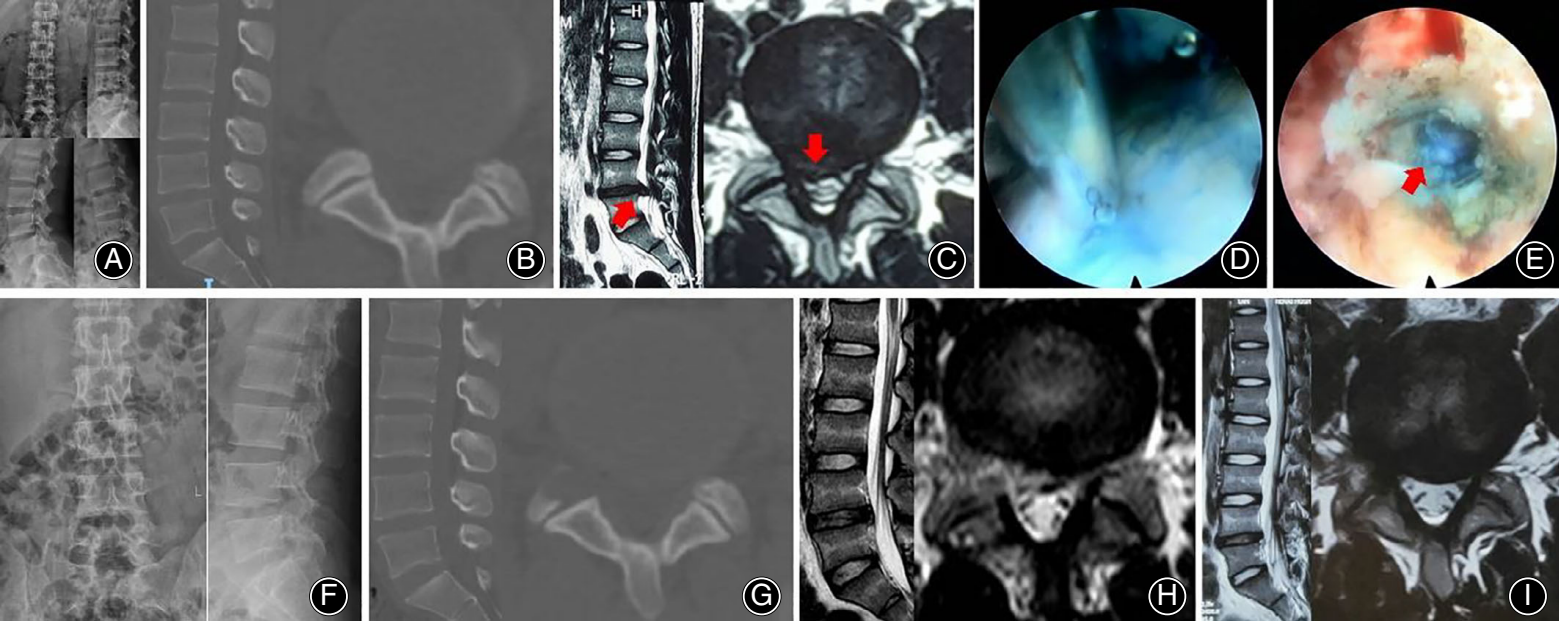
A 22‐year‐old male patient suffered from right lower extremity pain for 2 years and underwent L4‐5 PELD surgery. (A, B, and C) Preoperative lumbar spine X‐ray, CT, and MRI. His diagnosis was L4‐5 LDH (arrow). (D and E) The process of endoscopic staining. (F, G, and H) Postoperative lumbar spine X‐ray, CT, and MRI. MRI showed that the herniated disc has been removed completely. Postoperative ODI value 8% value and VAS score (leg pain 0 score) were significantly lower than preoperative ODI (42%) and VAS (leg pain 6 score). (I) Lumbar MRI images at 6 months follow‐up showed annulus fibrosus self‐rehabilitation.

Then, a methylene blue mixture of (Jumpcan Pharmaceutical Co., China) 1 mL + 0.9% N·S 9 mL) was injected under endoscope, which could see the mixture overflowing from rupture of the annulus fibrosus, accompanied by some degenerative nucleus pulposus (blue staining). Sometimes the nucleus pulposus was rushed out due to the increased pressure on the disc. Forceps can be used to remove protrusive NP and decompressed nerve root (Fig. 4A–D, Fig. 5 surgical diagrams, and Supplemental material S1). Finally, the surgeon adjusted the endoscopic view in different orientations to avoid any disc residuals, and verified the nerve root was decompressed fully. Dural sac became flat when the nerve root was completely decompressed (Fig. 6). Intraoperative blood loss less than 10 mL. Herniated disc decompressed was confirmed by postoperative MRI (Fig. 7, 8 and 9). All the preoperative symptoms were recovered completely after 2 days after surgery. The length of hospital stay was 5 days. Postoperative Oswestry Disability Index (ODI) value (14% value) and visual analog scale (VAS) score (1 score) were significantly lower than preoperative ODI (52%) and VAS (6 score).

## Discussion

### Surgical Approaches for LDH

LDH is a common cause of lower back pain and leg pain/numb, and incidence of which is increasing in all age groups. In the past, Posterior open discectomy, especially microdiscectomy, has been the standard surgery for treating LDH since the 1960s, but PELD has evolved rapidly over the last 30 years^18^. Since the first report of posterolateral endoscopic discectomy in 1992, it has been widely used to treat patients with uncontained lumbar disc herniation which has been extensively used by spine surgeons around the world. Currently, PELD is growing in popularity for the treatment of disc herniation^19^, ^20^, with advances in instrumentation including endoscopes, trephine and side‐firing. Especially the application of nucleus pulposus staining, which ensures a good curative effect, and improves the efficiency of PELD^21^, ^22^, ^23^, ^24^, ^25^. Some studies showed it also reduces the damage to the lumbar dorsal muscles, facet joints, ligament flavum and other soft tissues, at the same time, make the surgery safer and faster^26^, ^27^.

### Advantage and technique points of disc staining under endoscope

Fluoroscopy‐guided discography was often combined with nucleus pulposus staining or without nucleus pulposus staining in previous percutaneous endoscopic surgery, which leads to the injection amount of methylene blue cannot be accurately controlled during the PELD^28^, ^29^. Excessive injection may result in staining of nerve roots, blood vessels, toxic effect, etc., which can be easily injured in the operation while a tiny injection or no staining may affect the surgical process^30^, ^31^, ^32^.

Endoscopic nucleus pulposus staining is an original technique based on traditional disc staining. This technique is also used in addition to routine disc herniation, which can also be used in cases of disc migration with ruptured disc fragments migrated upward and downward in the spinal canal. The surgeon could find a large annular defect in the central annular zone and snatch the migrated disc fragments through the annular defect with the help of endoscopic staining with methylene blue injection. The range and direction of methylene blue staining could be clearly displayed under the endoscopy, and the rupture of the annulus fibrosus and the stained degenerative herniated nucleus pulposus could be found quickly and accurately according to the leaking position of the stain, which lead to the operative time being significantly reduced and the rate of nucleus pulposus omission was decreased. It is possible that the degenerative and herniated nucleus pulposus cannot be completely removed during the operation, resulting in the omission of residual nucleus pulposus. Postoperative symptoms cannot be alleviated, and the surgical effect is not good. In these cases, we excised all stained loose nucleus pulposus, which is considered as an indicator for decompression.

Due to the visualization of the whole endoscopic nucleus pulposus staining, the endoscopic staining group can accurately and efficiently complete the exploration of the Intervertebral disc rupture, which could reduce the operative time in PELD, and protect the undegenerated lumbar disc to the maximum extent. In addition, it has been reported in the literature that the short‐term recurrence rate of PELD is 4%–10%^33^. The causes of short‐term recurrence include age, previous diseases, degree of disc degeneration, selection of surgical indications, lack of surgical experience, unskilled operative technique and intraoperative over‐damage of intervertebral discs^7^, ^17^, ^34^. In addition to the good postoperative rehabilitation training, another important factor was that the intraoperative invasion of the undegenerated annulus fibrous tissue was less, which reduced the iatrogenic damage of the undegenerated intervertebral disc tissue to the greatest extent.

Therefore, this modified technique helps decrease the operation time, tissue injury degree, and improves the efficacy as well as safety. In addition, the learning curve is smooth and it is easy to master this technique for experienced orthopedic and neurosurgical spine specialists.

### Limitation

Several limitations of this study should be acknowledged. The number of included subjects was relatively small. However, we only aimed to introduce this modified technique of femur reconstruction and the modified technique of endoscopic staining in percutaneous endoscopic lumbar discectomy. Our final assessment of healing was acceptable given the radiographic appearances as well as absence of symptoms. We will include larger samples for retrospective and prospective clinical studies to further evaluate the definitive efficacy.

### Conclusions

Endoscopic staining in PELD can minimize iatrogenic injury of undegenerated annulus fibrosus and more quickly complete the nerve root decompression. It could be a supplemented technique in PELD surgery.

## Supporting information


**Appendix** **S1** Supplementary Information.
